# High-Resolution and Specific Detection of Bacteria on Complex Surfaces Using Nanoparticle Probes and Electron Microscopy

**DOI:** 10.1371/journal.pone.0126404

**Published:** 2015-05-27

**Authors:** Jun Ye, Shaun Nielsen, Stephen Joseph, Torsten Thomas

**Affiliations:** 1 Centre for Marine Bio-Innovation, School of Biotechnology and Biomolecular Sciences, The University of New South Wales, Sydney, Australia; 2 School of Materials Science and Engineering, The University of New South Wales, Sydney, Australia; University Hospital of the Albert-Ludwigs-University Freiburg, GERMANY

## Abstract

The study of the interaction of bacteria with surfaces requires the detection of specific bacterial groups with high spatial resolution. Here, we describe a method to rapidly and efficiently add nanogold particles to oligonucleotide probes, which target bacterial ribosomal RNA. These nanogold-labeled probes are then used in an *in situ* hybridization procedure that ensures both cellular integrity and high specificity. Electron microscopy subsequently enables the visualization of specific cells with high local precision on complex surface structures. This method will contribute to an increased understanding of how bacteria interact with surface structures on a sub-micron scale.

## Introduction

The study of interactions between microorganisms and biotic or abiotic surfaces is fundamental to the field of microbial ecology, with the spatial detection and description of such interactions receiving much attention in recent years [1, 2]. Surface structures, such as redox-active mineral deposits or organic materials with sub-micron dimensions, are thought to strongly influence the ability of specific microorganisms to colonize and proliferate upon surfaces, which leads to the heterogeneous structure and composition of microbial communities [3, 4]. However to fully understand these interactions require that specific microorganisms can be localized on surfaces with high spatial resolution.

Fluorescence *in situ* hybridization (FISH), which employs fluorescently-labeled oligonucleotide probes to target ribosomal RNA (rRNA), is arguably the most widely used method to detect specific bacteria and their spatial arrangement in environmental samples [5, 6]. However, the application of FISH to complex environmental samples is often limited by the theoretical optical resolution of light microscopy (~0.25 μm) [7] and the background autofluorescence of certain type of samples, such as soil particles, tissues and mineral surfaces [8, 9].

Electron microscopy (EM) can overcome these issues and provides highly resolved localization (to the nanometer scale) of microbial cells with minimal background interference [10]. Specific microorganisms can be detected with EM by replacing the fluorescent labels used in FISH with nanoparticles of gold (nanogold), which are then detected via backscattered electrons (BSE) [11]. Gold *in situ* hybridization (GISH) approaches have recently been employed using three different strategies to detect specific microorganisms [12–15]. Firstly, oligonucleotide probes that were physically linked to an antigen were detected post-hybridization by antibody-nanogold conjugates [12]. Secondly, biotin-labeled oligonucleotide probes were detected using streptavidin-nanogold conjugates [13, 14]. And thirdly, oligonucleotide probes coupled to horseradish peroxidase (HRP) were used to deposit biotinylated tyramide inside the cell, which was subsequently detected with streptavidin-nanogold conjugates [15]. A drawback of all these approaches, however, is that they require large biomolecules (i.e. HRP, antibody, streptavidin) to enter cells, which requires permeabilization of the cell membrane. This can lead to the leakage of rRNA from the cell and hence to signals with sub-optimal spatial resolution as been pointed out by Schmidt et al. [15]. In addition, there is no comprehensive method for the inactivation of endogenous peroxidases in various sample types and this can cause false-positive signals during HRP-mediated tyramide deposition [16].

Recently, Kubota *et al*. [17] reported that oligonucleotide probes, already coupled to a nanogold particle by a commercial provider (Tsukuba Oligo Service), could be used to detect specific bacteria using nano-secondary ion mass spectrometry (NanoSIMS). Hybridization was conducted without the need of cell permeabilization owing to the small size of the nanogold-oligonucleotide conjugate. This direct coupling strategy is promising as it could potentially overcome the “signal leakage” problem of the other GISH methods mentioned above, however this requires evaluation by high-resolution EM.

Here we present a simple method to label a standard 5’ thiol-modified oligonucleotide probe with nanogold particles (average diameter of 1.4 nm) and then apply this nanogold-oligonucleotide conjugate in an *in situ* hybridization protocol without permeabilization. We then demonstrate the sensitivity of detection for specific bacteria with high spatial resolution on complex (three-dimensional) surface structures using scanning electron microscopy (SEM).

## Materials and Methods

### Model system

To develop our methodology we used two morphologically distinct bacteria: rod shaped *Escherichia coli* JM109 (class *Gammaproteobacteria*) and coccoid *Neisseria sicca* (class *Betaproteobacteria*). Both cell types were cultured separately at 37°C in Mueller Hinton broth (MHB) and harvested (2 ml) at logarithmic phase by centrifugation (5000 x g, 10 min). The pelleted cells were washed twice in phosphate buffered saline (PBS, 20 mM NaH_2_PO_4_, 150 mM NaCl, 1 mM EDTA, pH 6.5) and finally resuspended in 750 μl PBS. Cells were fixed by adding 250 μl of 4% (v/v) paraformaldehyde and incubated at 4°C for 16 h. Cells were again pelleted and washed twice in PBS and stored at—20°C in 1 ml of a 1:1 mixture of PBS and absolute ethanol.

To detect specific bacteria on a complex surface structure we used biochar particles inoculated with a mixture of *E*. *coli* and *N*. *sicca*. Briefly, biochar was produced from bamboo stems, kaoline and iron mineral by pyrolysis at 450°C. The particle size fractions between 2 mm and 5 mm were selected and dried at 70°C for 2 h. Biochar particles were pre-wetted with 2 ml MHB for 24 h and excess liquid was subsequently removed with sterile absorbent paper. Biochar particles were then inoculated with 200 μl of a 1:1 mixture of *E*. *coli* and *N*. *sicca* and incubated at 37°C for 3 days to facilitate surface colonization and cell proliferation. Biochar samples were finally fixed and stored as described for the cell cultures (see above).

### Nanogold-oligonucleotide probe preparation

The oligonucleotide probe EUB338 (5’ GCT GCC TCC CGT AGG AGT 3’) targeting the 16S rRNA molecules of *Bacteria* [18] and GAM42a (5’GCC TTC CCA CAT CGT TT 3’) targeting the 23S rRNA molecules of the class *Gammaproteobacteria* were used [19]. The oligonucleotide probe was modified at the 5’ terminus with a free thiol group (Biorise, Australia). As free thiols are easily oxidized to form disulfide bonds, 100 μM modified probe were incubated in 100 mM mercaptoethylamine hydrochloride (MEA) at 48°C for 90 min to reduce the disulfide bonds. An Amicon centrifugal filter (Millipore, USA) with a cut off size 3 kDa was then used to separate the reduced probe from the MEA. Three micromolar of reduced probe was coupled with 30 μM monomaleimido nanogold (Nanoprobes, USA) at 4°C for 24 h in PBS buffer. The nanogold-oligonucleotide probe was then purified from unbound nanogold with either standard ethanol precipitation, or a Micro Bio-spin P-6 chromatography column (Bio-Rad, USA) following the manufacturer’s instruction. Purified conjugates were then stored at 4°C until use. Gel electrophoresis was used to analyze the efficiencies of the coupling and purification processes. Since nanogold quenches fluorescence dyes [20], the same volume of sample was loaded on two identical 20% non-denaturing polyacrylamide gels and run simultaneously at a constant 200 V for 120 min using a Mini PROTEAN 3 System (Bio-rad, USA) that holds two gels. The gels were then treated either with LI SILVER (Nanoprobes) to detect the unbound nanogold and nanogold-oligonucleotide conjugates or with SYBR GOLD (Life Technologies) to stain the unlabeled oligonucleotides. The gels were visualized with a Gel Doc XR+ system (Bio-rad, USA) and images were superimposed. Reaction and recovery efficiencies were calculated with GelEval (Frogdance, UK).

### Gold *in situ* hybridization (GISH)

Fixed cells of cultured *E*. *coli*, *N*. *sicca*, the mixture of both bacteria, as well as cells added to biochar (see above) were hybridized with the nanogold-oligonucleotide conjugates as follows. Either 20 μl of fixed cells were applied onto a circle cover slip (15 mm) or one biochar particle was transferred to a tube (0.5 ml). Samples were left to air dry and then dehydrated in sequential 3 min immersions of 50%, 80% and 100% ethanol. *In situ* hybridization was accomplished using 50 μl hybridization buffer (900 mM NaCl, 20 mM Tris/HCl, 35% deionized formamide, 0.01% SDS) containing 10 μl purified nanogold-oligonucleotide conjugate (5.5 ng oligonucleotide μl^-1^) at 46°C for 3 h. Removal of unbound probe was performed by consecutive washes in PBS (three times for 10 min at room temperature), and once in pre-warmed washing buffer (70 mM NaCl, 20 mM Tris/HCl, 5 mM EDTA, 0.01% SDS) for 30 min at 48°C. A negative control was treated the same way, except that no nanogold-oligonucleotide probe was used.

### Gold enhancement

Given that the diameter of nanogold (1.4 nm) used in this study is below the resolution limits of SEM (5 nm), we performed a gold enhancement step to increase the size of the nanogold after hybridization. This involved depositing soluble gold ions onto the primary nanogold coupled to the oligonucleotide and enlarging it to a detectable size of up to 20 nm [21]. Cover slips or biochar particles were first washed three times in PBS for 10 min each at RT and then three times in MilliQ water for 10 min each at RT. Both PBS and MilliQ water were previously filtered through a 0.22 μm sterile polyethersulfone membrane (Millipore, USA). GOLDENHANCE EM Plus (Nanoprobes, USA) was used to metallographically enlarge nanogold particles. Briefly, 40 μl of each of “enhancer” solution and “activator” solution was mixed and incubated for 5 min at RT before mixing with the same volume of each of the “initiator” solution and the “buffer” solution. These solutions were thoroughly mixed and immediately dropped onto cover slips and biochar particles (ensuring complete emersion). Signal enhancement was stopped by rinsing the sample three times for 10 min each in MilliQ water,

### Scanning electron microscopy (SEM) and energy dispersive X-ray spectroscopy (EDS)

Samples were dehydrated in a series of ethanol and hexamethyldsilazane (HMDS) solution (Sigma, Australia) as follows: 50%, 70%, 80%, 90%, 95%, 100% ethanol, 2:1 ethanol/HMDS, 1:1 ethanol/HMDS, and 100% HMDS for 10 min each [22]. Subsequently, dehydrated samples were mounted on stubs with self-adhesive pads and coated with evaporated carbon layer (JEE-420 Evaporative Carbon Coater, USA). Images of secondary electrons (SE), backscattered electrons (BSE) were generated with a HITACHI S-3400 SEM (Hitachi, Japan) using the respective detectors. X-ray elemental analysis was conducted by using XFlash 6 detector (Bruker, UK) integrated with HITACHI S-3400 SEM. EM was performed under high-vacuum mode, the working distance of detectors from the specimen was set to 10 mm and the accelerating voltage was applied at 15 kV for both SE and BSE images and 20 kV for EDS images.

Cell counting was performed manually in ImageJ by using the cell counter function and only enumerating cells that were either clearly rod-shaped or round. Ten images at 6000x magnification were randomly selected for each sample and the detection rate was calculated as the proportion of cells with signal under the BSE mode compared to the total cells (i.e. SE mode).

## Results and Discussion

### Synthesis and purification of nanogold-oligonucleotide conjugates

**Fig 1:**
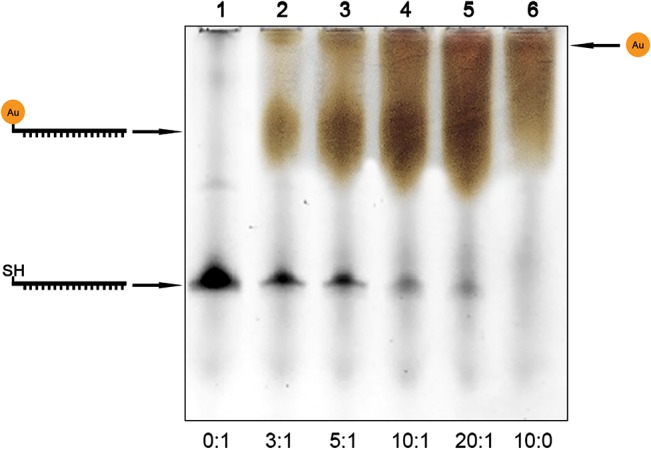
Polyacrylamide gel electrophoresis of unlabeled oligonucleotide (lane 1), products of oligonucleotide and nanogold coupling at different molar ratios (lanes 2–5) and unbound nanogold (lane 6). Molar ratios of nanogold to oligonucleotide are given at the bottom. The arrows indicate positions of the unbound nanogold (top, brown smear), nanogold-oligonucleotide conjugates (middle, brown bands), and unlabeled oligonucleotide (bottom, black bands), respectively. Gels were imaged separately and subsequently superimposed to obtain the final composite image.

To develop and optimize a method for the production of nanogold-oligonucleotide conjugates, we first tested the reaction of nanogold and oligonucleotide in different molar ratios. Gel electrophoresis was used to analyze the coupling of nanogold to oligonucleotide, as it should produce a significant shift in electrophoretic mobility [23]. As shown in Fig 1, when no nanogold was present, a single SYBR GOLD-stained band corresponding to the oligonucleotide was visible (Lane 1, bottom arrow). Nanogold particles, which have limited charge [24], barely migrated into the gel (top smear in lane 6). With increasing ratios of nanogold to oligonucleotide (lane 2–5) the SYBR GOLD signal for the unlabeled oligonucleotide diminished and instead increasingly pronounced smears appeared in the position between the unlabeled oligonucleotide and the nanogold (middle arrow). This showed the expected outcome that the oligonucleotide was coupled as a function of increasing amounts of nanogold, and that the migration of the oligonucleotide was retarded when bound to a nanogold.

Neither UV/visible spectroscopy nor mass-spectrometry were found to be accurate to quantify the nanogold-oligonucleotide conjugate (data not shown). Conjugation efficiencies were therefore calculated by comparison of band intensities with the unlabeled oligonucleotide. Efficiencies were found to be 51%, 56%, 72% and 76% for 3:1, 5:1, 10:1 and 20:1, respectively. To maximize the production of desired nanogold-oligonucleotide conjugate and minimize the remainder of uncoupled nanogold, the reaction ratio of 10:1 was applied in the following experiments.

**Fig 2:**
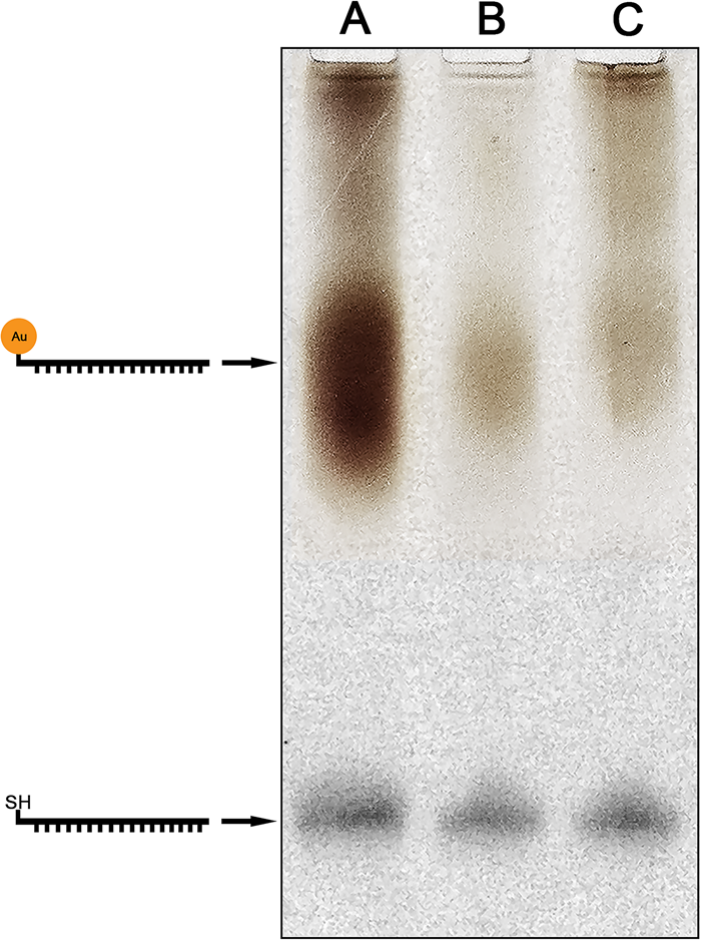
Polyacrylamide gel electrophoresis of nanogold-oligonucleotide conjugates before (lane A) and after purification using gel filtration (B) or ethanol precipitation (C). Gels were imaged separately and subsequently superimposed to obtain the final composite image. Brown bands represent nanogold-oligonucleotide conjugates. Black bands represent unlabeled oligonucleotide.

For the purification of the nanogold-oligonucleotide conjugate, gel filtration (Fig 2, lane B) and ethanol precipitation (Fig 2, lane C) were tested. There was little difference for both methods in terms of recovery efficiency of the conjugate, with typical yields between 25–30%. Similar recovery rates were also observed previously using glassfiber microfilters to isolate conjugates of double-stranded DNA and 10 nm nanogold [25]. However, in our experience gel filtration removed more of the unbound nanogold providing higher purity of the nanogold-oligonucleotide conjugate (Fig 2, lane B), which resulted in a cleaner background when applied to *in situ* hybridization (data not shown).

### Detection of cells and gold enhancement

**Fig 3:**
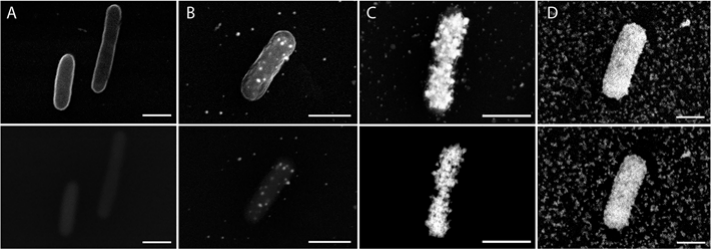
Electron micrographs of GISH for *E*.*coli* cells after different times of gold enhancement by GOLDENHANCE EM Plus (Nanoprobes). Panels A-D correspond to 0 min, 1.5 min, 3 min, 6 min of gold enhancement, respectively. The top and bottom panels are shown for SE and BSE images, respectively. Scale bar = 1 μm.

We first applied a nanogold-labeled GAM 42a probe without gold enhancement to detect pure *E*. *coli* cells on a microscope slides. Under SE mode cells were clearly visible, but no signal was observed using BSE detection (Fig 3A). This was expected as the bound 1.4 nm nanogold particle was below the detection limit of the SEM used here. We therefore increased the nanogold particle size by a metallographic deposition of soluble gold ions. As the reaction time for the gold enhancement increased, signal intensity using BSE detection increased (Fig 3B). We noted that these signals were well defined within the perimeter of the cells and that each dot might represent a binding event to single intracellular rRNA molecule. We found that enhancement times of approximately 3 min for cells on cover slips (Fig 3C) and 2 min for cells on biochar surface were optimal for obtaining sufficient signals for cell detection and low background noise. However we recommend testing a range of enhancement times for other sample types. Excessive gold enhancement increased background noise, which was not concentrated near the cell, but rather evenly spread across the surface (Fig 3D). The lack of background noise near the cells indicates that they remained intact and did not release cellular contents, such as rRNA.

### Specificity of GISH protocol

**Fig 4:**
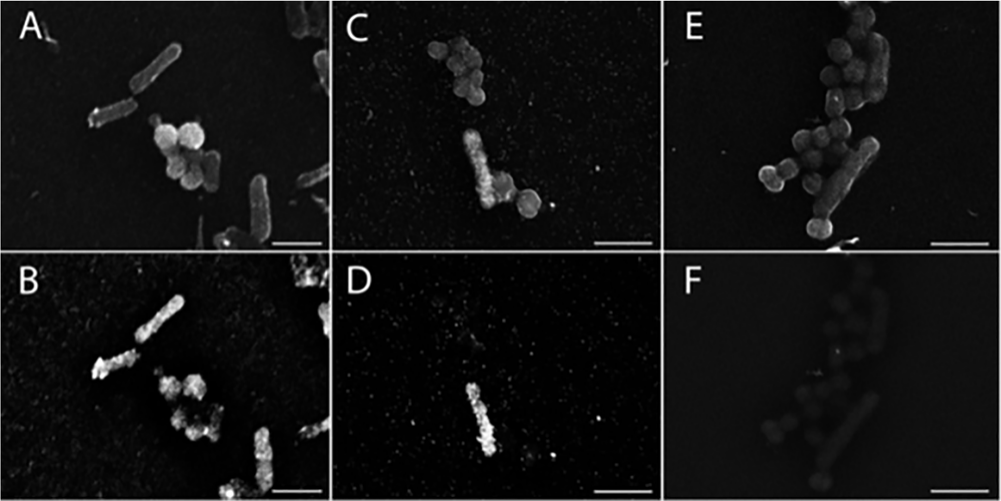
Electron micrographs of GISH for mixtures of *E*.*coli* (rod) and *N*.*sicca* (cocci) cells using different nanogold-oligonucleotide probes: EUB338 (A and B), GAM 42a (C and D), gold enhancement without probes (E and F). The same microscopic fields are shown with SE (top, A, C, E) and BSE (bottom, B, D, F) images. Scale bar = 2 μm.

Next we tested the specificity of the GISH method on a mixture of rod-shaped *E*. *coli* and coccoid *N*. *sicca* using either the general-bacteria probe EUB338 or the probe GAM 42a (Fig 4), which only targets gamma-proteobacteria like *E*. *coli*. The two different cell types were clearly recognizable under SE mode (Fig 4A, 4C and 4E). Hybridisation with probe EUB338 gave clear signals for both cell types in the BSE mode, showing that that 1.4 nm nanogold-labeled probe could enter *E*.*coli* and *N*.*sicca* without any additional permeabilisation. This BSE signal was not due to unspecific effects of the gold enhancement, as a GISH treatment without the nanogold-labeled probe, but including the gold enhancement step, showed no signal for both type of cells (Fig 4E and 4F).

When the specific GAM 42a probe was used, many electron-dense signals were visible in the SE mode for *E*. *coli*, but not *N*. *sicca*, (Fig 4C). This difference became even more apparent under BSE mode, where the outline of rod-shaped *E*. *coli* cells were clearly seen, but *N*. *sicca* cells were practically invisible (Fig 4D). The GISH signals were clearly defined even when *N*. *sicca* cells were immediately adjacent to *E*.*coli* cells highlighting again the lack of extracellular, false-positive signals, and also the high spatial resolution obtained. We also note that the GAM 42a probe was identical to the rRNA gene sequence of *N*. *sicca* [19] except for a single mismatch, yet no false-positive signal was obtained. Together these results demonstrate the high specificity of the nanogold-labeled probe and our GISH protocol to detect bacterial cells of interest.

### Application of GISH to complex surfaces

To test the applicability of our GISH protocol to complex surfaces, we colonized biochar particles with a mixture of *E*. *coli* and *N*. *sicca*. Biochar is a complex, porous material with many sub-micron structures of mineral and organic matter and this may provide suitable habitats for microorganisms by providing spatially defined nutrient or energy resources [26]. However, the structural and chemical complexity of biochar limits its analysis with fluorescence microscopy, both in terms of optical resolution and fluorescence from humic and aromatic compounds found in biochar [27]. Biochar thus represents a suitable test case to assess the applicability of our method to a complex surface, such as those found in the natural environment e.g. soil particles [28] or host tissue [29].

**Fig 5:**
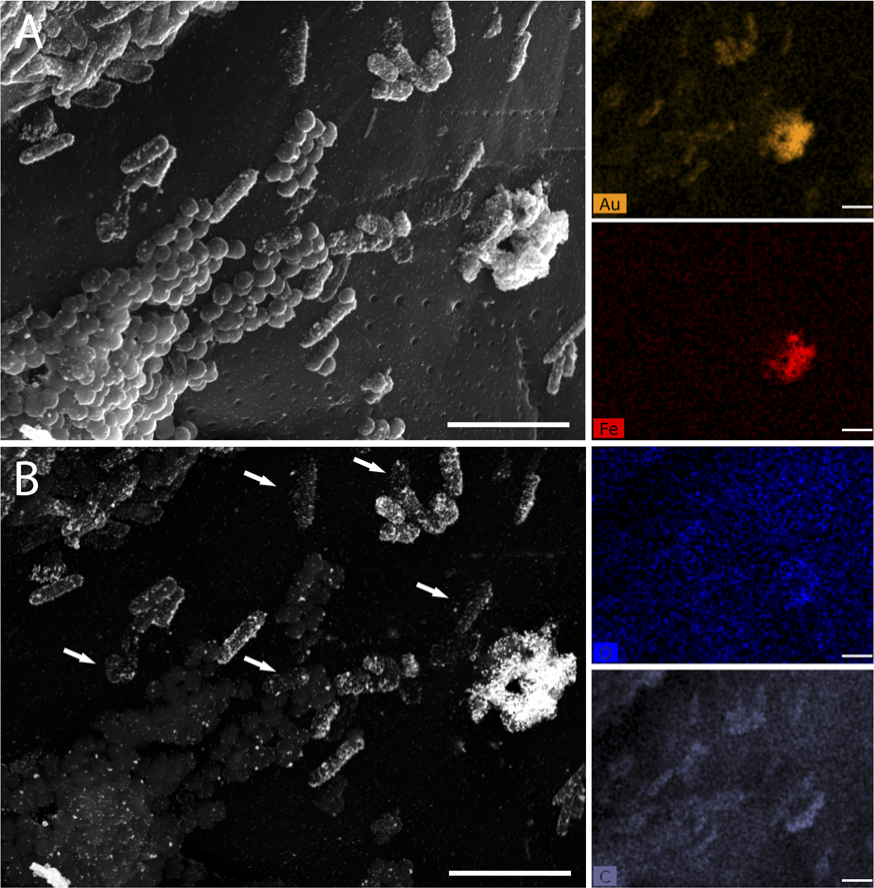
Electron micrographs of GISH for mixtures of *E*. *coli* (rod) *N*. *sicca* (cocci) cells on a biochar surface. Panel A and B correspond to SE image and BSE image, respectively. Arrows indicate positions of *E*.*coli* cells with low GISH signals. The panel on the right part corresponds to different element maps of the energy dispersive X-ray spectroscopy (EDS): Au (yellow), Fe (red), O (blue), and C (purple). Separate and larger EDS images are available as S1–S4 Figs Scale bar = 2 μm.

After incubating *E*. *coli* and *N*. *sicca* cells with biochar and performing GISH with the nanogold-GAM 42a probe, we obtained high-resolution images of both bacterial cells and surface structures (Fig 5A). As above, *E*. *coli* cells could be clearly distinguished from *N*. *sicca* in the BSE mode (Fig 5B). The electron-dense signal was weaker for some *E*. *coli* cells (see arrows in Fig 5B), when compared to exponentially-grown cells (see Fig 4A) and this is likely due to the 3-day incubation on the biochar resulting in cells at different growth stages and hence different rRNA content [30]. Nevertheless, the detection rate for our GISH protocol when applied to biochar structures was 69.4 ± 8.4%, which was similar to the rate (60–75%) reported by Schmidt *et al*. for a GISH method employing a HRP-mediated tyramide signal amplification [15].

In the BSE mode we also noted large electron-dense aggregates in certain locations of the biochar surface (Fig 5B) that gave strong gold signals with energy dispersive X-ray spectroscopy (EDS). These signals could potentially be due to *E*. *coli* cells clumping together, however, the aggregates also had strong Fe and O signals indicating that they consist of iron oxides. These would have functioned as a nucleus for the deposition of soluble gold ions during the gold enhancement. In contrast, *E*. *coli* cells lacked the Fe signal, but instead showed cell-shaped carbon signals in the EDS (Fig 5). Therefore, EDS can be potentially used to further distinguish the true-positive detection of bacterial cells from the false-positive signals that may come from metal elements that might be present in natural samples [14]. However some bacteria can also accumulate metal particles [31], which could complicate such an interpretation of the EDS signal.

## Conclusions

Although DNA has been previously labeled with nanogold [32], we present here an efficient and simple method to couple nanogold particles to standard 5’ thiol-modified oligonucleotides that are readily used for *in situ* hybridization. Due to the small size of the nanogold-oligonucleotide conjugates and the post-hybridization signal amplification with metallic gold, our GISH protocol requires no cell permeabilization, which avoids cell-derived background signals. Ultimately, this results in a) highly resolved and b) specific localizations of target cells, even on complex surface structures. While we have here only shown the specific detection of bacteria in a simple test system, we are confident that these two features will make our protocol suitable for the detection of specific bacteria (or bacterial groups) in more complex communities. Combined with EDS (or other analytical techniques) this can link the position of specific bacteria with surface structures that may be important for bacterial metabolism, such as organic material or mineral depositions used as electron acceptor or donors [3, 4]. Our method has therefore the potential to elucidate the interactions of specific bacteria and their environment on a very small scale.

## Supporting Information

S1 Fig Elemental map of energy dispersive X-ray spectroscopy (EDS) for Au. (TIF)

S2 Fig Elemental map of energy dispersive X-ray spectroscopy (EDS) for Fe. (TIF)

S3 Fig Elemental map of energy dispersive X-ray spectroscopy (EDS) for O. (TIF)

S4 Fig Elemental map of energy dispersive X-ray spectroscopy (EDS) for C. (TIF)
