# Supplementary figures and images for: High-Resolution and Specific Detection of Bacteria on Complex Surfaces Using Nanoparticle Probes and Electron Microscopy
Source: PLoS One. 2015 May 27;10(5):e0126404. doi: 10.1371/journal.pone.0126404 (PMC4446341; doi:10.1371/journal.pone.0126404)

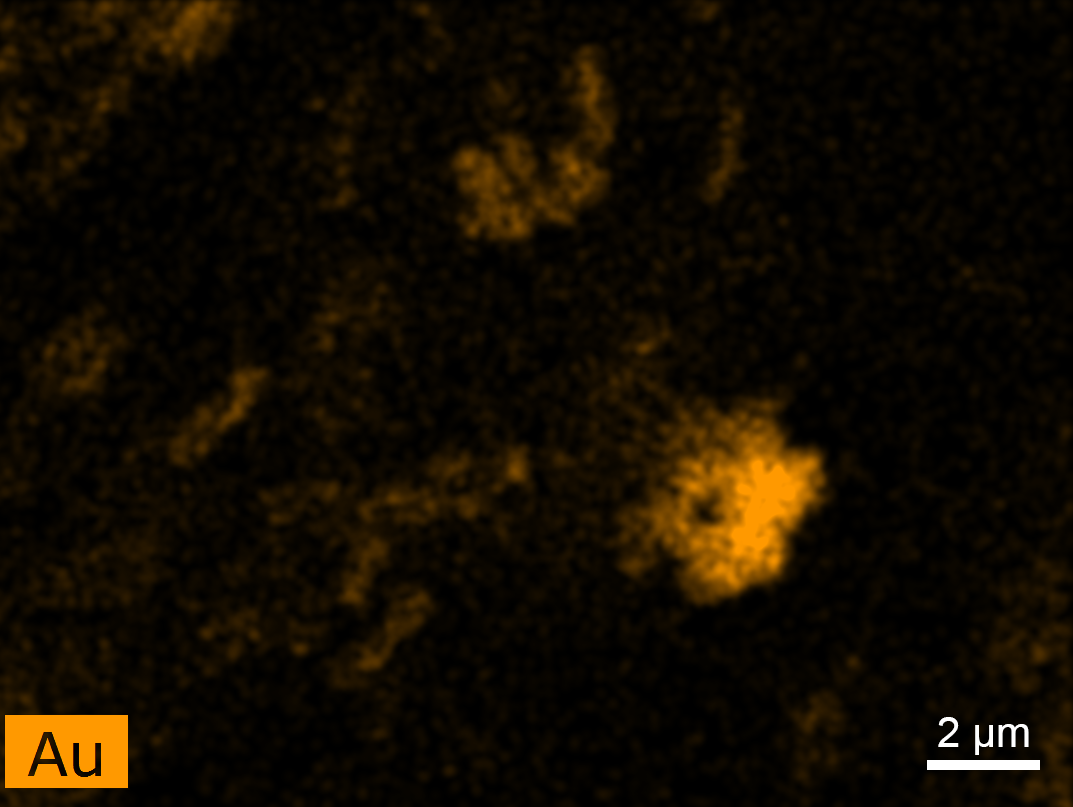

Supplement: S1 Fig — (TIF) [file pone.0126404.s001.tif]

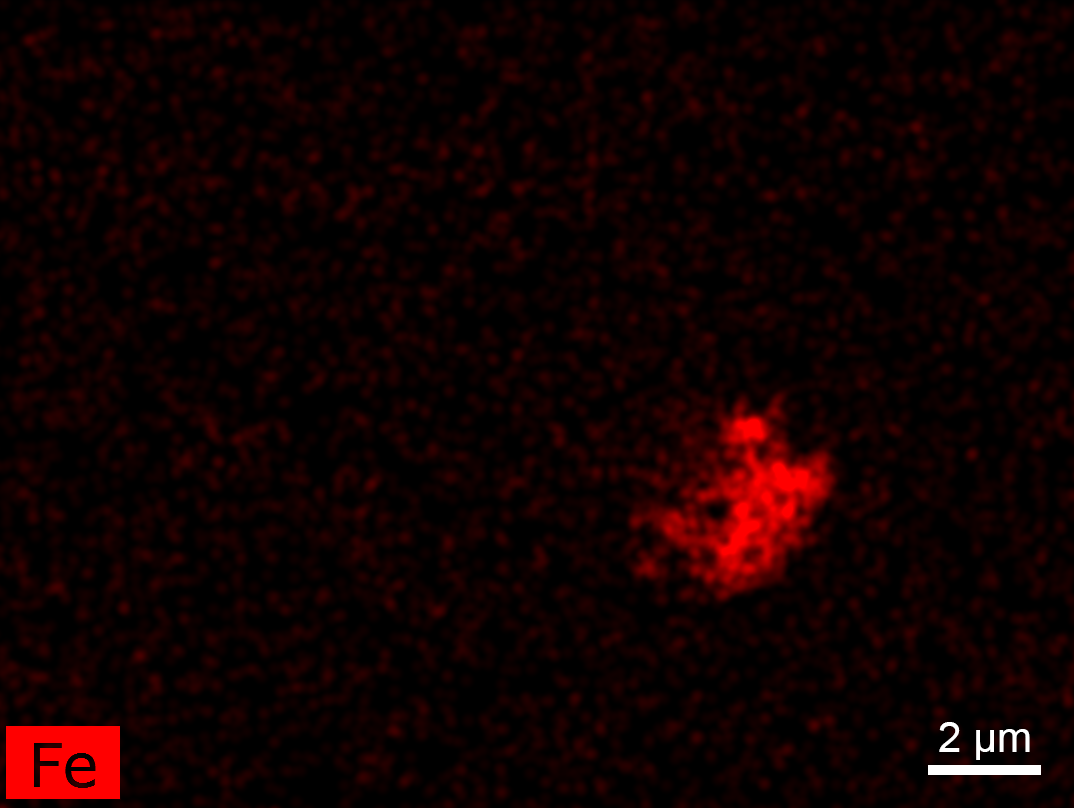

Supplement: S2 Fig — (TIF) [file pone.0126404.s002.tif]

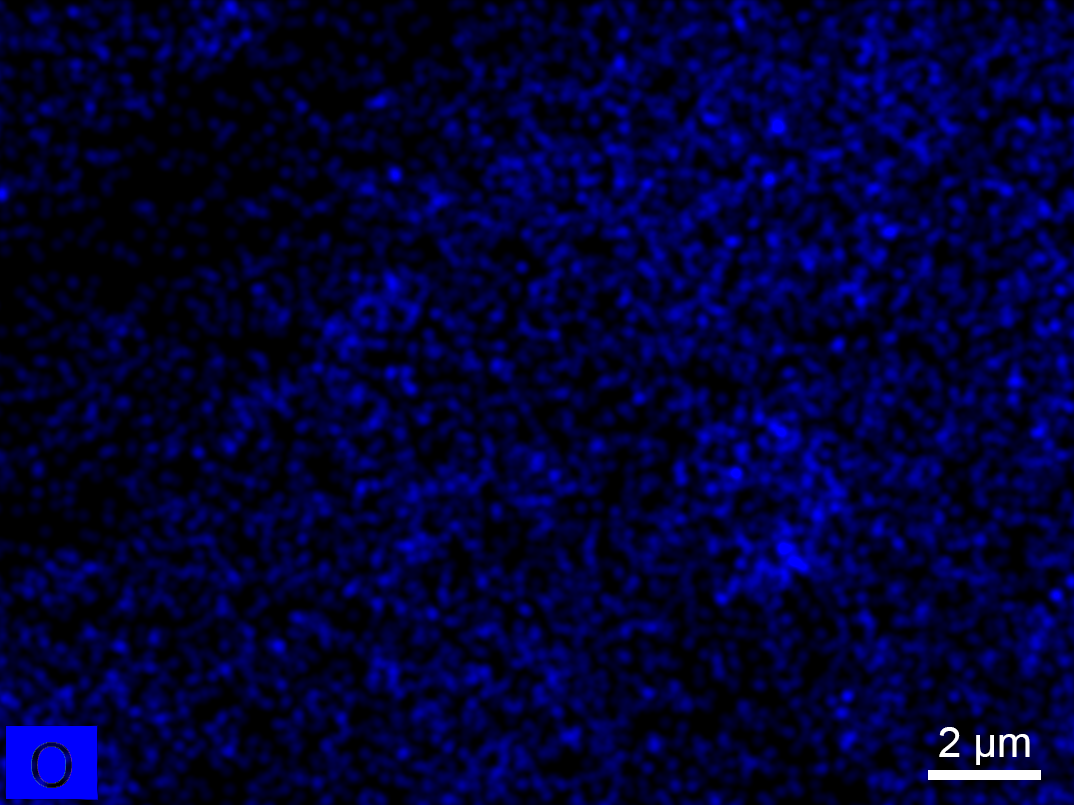

Supplement: S3 Fig — (TIF) [file pone.0126404.s003.tif]

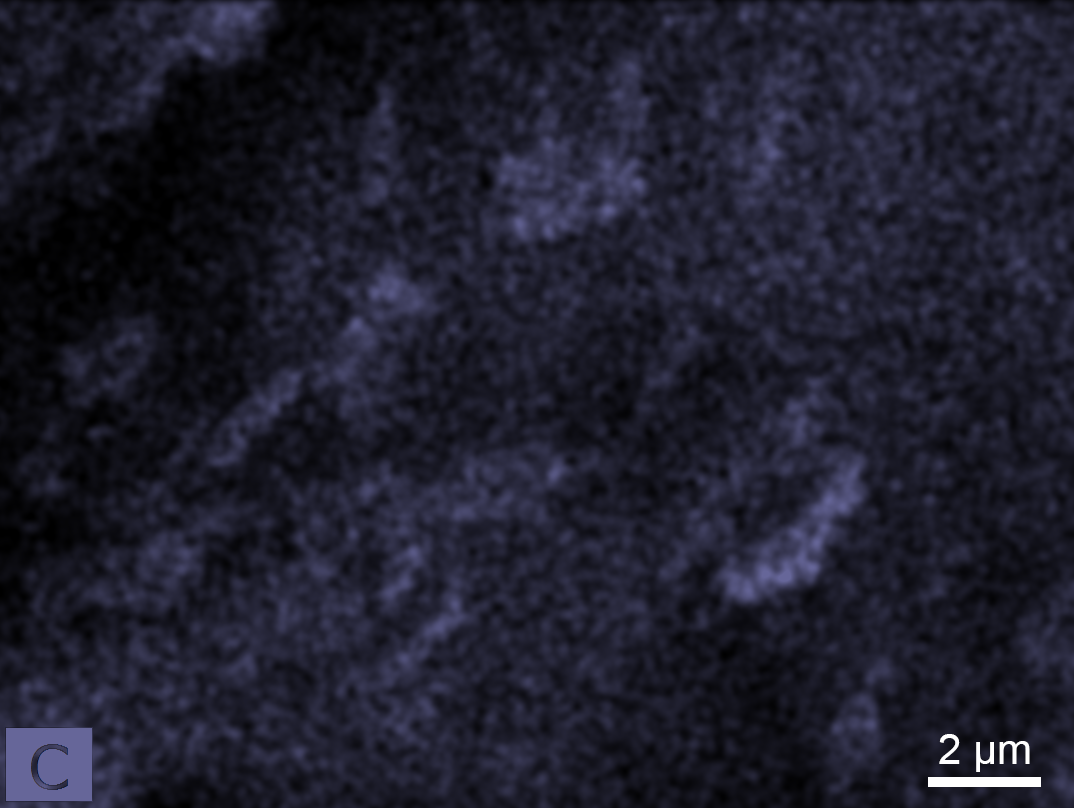

Supplement: S4 Fig — (TIF) [file pone.0126404.s004.tif]
